# Histopathological Assessment and In Ovo Vaccination Response to IBD and ND in Broiler Chickens

**DOI:** 10.3390/ani15121722

**Published:** 2025-06-11

**Authors:** Marcin Wegner, Adrian Żurek, Joanna Frischke-Krajewska, Michał Gesek

**Affiliations:** 1 Boehringer-Ingelheim, 00-728 Warsaw, Poland; marcin.wegner@op.pl (M.W.); adrian.rzurek@gmail.com (A.Ż.); 2 Vetdiagnostica Sp. z o.o., Otorowo 30, 86-050 Solec Kujawski, Poland; krzysztof@vetdiagnostica.com; 3 Department of Pathological Anatomy, Faculty of Veterinary Medicine, University of Warmia and Mazury in Olsztyn, 10-719 Olsztyn, Poland

**Keywords:** broiler, Gumboro antibody titer, bursa of Fabricius, Newcastle disease

## Abstract

Infectious bursal disease (IBD) is a viral disease that most commonly affects young chickens and destroys lymphocytes, leading to immunosuppression. The field study aimed to investigate the effect of three different vaccines administered in ovo against IBD and spray against Newcastle disease (ND) on serological response tested for IBD and ND and histopathological analysis of the bursa of Fabricius (BF) and quantitative B lymphocytes in BF in broiler chickens. The study was conducted on a farm of four hen houses with 30,000 chicks in each building. Three different vaccination programs were used in the poultry hatchery, and one hen house IV was not vaccinated. All three groups were vaccinated at 18 days and 9 h in ovo during egg transfer against IBD at a dose of 0.05 mL/embryo, group I vector vaccine (strain vHVT013-69), group II immunocomplex vaccine (strain Winterfield 2512), group III immunocomplex vaccine (strain M.B, 0.05). Then, after hatching, the chicks were vaccinated in a spray (groups I, II, and III) against NDV (strain VG/GA, 20 mL/100 birds) and infectious bronchitis (IBV) in a spray (strain H-120, serotype Mass, and strain CR88121, serotype 793B) at a dose of 20 mL/100 chicks. On days 1, 21, 31, and 41, blood was collected for serological tests to determine the antibody titer against IBD, which was performed using two tests (IDEXX and ID-Vet) and against ND. During the necropsy of birds on days 21 and 31, the bursae of Fabricius were collected from five chickens for histopathological evaluation of BF and quantitative B lymphocyte counts; a total of 40 bursae were analyzed (10 per group). The vaccination program applied significantly (*p* < 0.05) affected the immune response expressed as a geometric mean titer (GMT) in the serum of the examined chickens against IBDV on days 21, 31, and 41. Differences were also demonstrated in the mass and level of BF damage and the number of B lymphocytes. No significant differences were demonstrated in the GMT in the serum of the examined chickens against NDV depending on the vaccination program applied.

## Simple Summary

Due to the increasing pressure of field viruses threatening birds during rearing, various preventive programs are used. The most dangerous viruses that attack broiler chickens include infectious bursal disease (IBD) and Newcastle disease (ND). The aim of the study was to investigate the effect of three different vaccines administered in ovo against IBD and spray against ND on the serological response tested for IBD and ND and histopathological analysis of the bursa of Fabricius (BF) and quantitative B lymphocytes in BF in broiler chickens. The vaccination program applied significantly (*p* < 0.05) affected the immune response expressed as a geometric mean titer (GMT) in the serum of the examined chickens against IBDV on days 21, 31, and 41. Differences were also demonstrated in the mass and level of BF damage and the number of B lymphocytes. No significant differences were demonstrated in the GMT in the serum of the examined chickens against NDV depending on the vaccination program applied.

## 1. Introduction

The ever-increasing production of broiler chickens is causing an increasing threat and risk of birds contracting various diseases. This is due to the very short rearing period (usually 42 days) and the number of production cycles per year. The increased risk of chickens contracting the disease is also influenced by mutations of viruses that occur in the environment and cause great pressure. Therefore, it is very important to follow and apply various preventive programs. In Poland, the most important diseases against which chicks are protected include Marek’s disease (MD), infectious bursal disease (IBD), Newcastle disease (ND), and infectious bronchitis (IB) [1]. One of the most dangerous viral diseases occurring in broiler chickens is IBD, which can manifest itself in high mortality but also lead to immunosuppression [2,3]. IBD virus belongs to the genus Avibirnavirus within the family Birnaviridae [2]. There are two serotypes: pathogenic (serotype 1) and nonpathogenic (serotype 2). The classical genotype (cIBDV) was first detected in 1957; then, in Gumboro County, Delaware, USA, the second genotype (varIBDV) was detected in 1987; and then highly virulent strains (vvIBDV) appeared in Europe in 1987 [4]. The disease most often affects young birds that are between 3 and 6 weeks of age because, at this time, the level of maternal antibodies is very low, causing high mortality, which can reach 60–70% with the vvIBDV strain [2,3,4,5]. Most often, the IBD virus causes damage to the bursa of Fabricius (BF), which is responsible for the production of B lymphocytes, causing permanent atrophy of the bursa [4,5]. Various prophylactic vaccinations are used to protect birds. Available vaccines against IBD are in live and inactivated form [2]. Depending on the pathogenicity of the viruses in the vaccine, they can be divided into mild, intermediate, intermediate plus, and hot [2,5]. Attenuated mild IBD vaccines break through maternally derived antibody (MDA) titers of 125, intermediate vaccines break through MDA titers of 250, intermediate-plus vaccines break through MDA titers of 500, and hot vaccines break through titers above 500 [5,6]. In contrast to mild vaccines, intermediate plus and hot vaccines break through higher levels of maternal antibodies, causing a faster immune response against IBDV. However, these vaccines also cause higher levels of immunosuppression, increasing the birds’ susceptibility to infection with other pathogens [7,8].

Newcastle disease (ND) is a highly contagious viral disease of poultry caused by the negative single-stranded enveloped RNA orthoavulavirus 1 (AOAV-1), which belongs to the family paramyxoviridae, subfamily paramyxovirinae [9,10]. Based on the pathogenic studies performed, the virus has been divided into asymptomatic (enteric), mesogenic, lentogenic, neutropic velogenic, and viscerotropic strains [9]. Depending on the strain (NDV) that causes the disease, mortality among chickens infected with the velogenic strain can reach up to 100% [9,11]. To protect birds against the virus, various vaccines containing lentogenic strains (Hitchner, B1, and LaSota) or apathogenic strains (Ulster and VG/GA) are used, which induce cellular and humoral immunity [9]. Despite the use of preventive programs aimed at protecting and limiting the spread of the virus, the ND virus continues to attack broiler chickens worldwide, causing large economic losses. The experiment aimed to investigate the effect of three different vaccines administered in ovo against IBD on the serological response tested for IBDV and NDV and histopathological analysis of BF and quantitative B lymphocytes in broiler chickens.

## 2. Materials and Methods

The research analysis (field study) was conducted on a commercial farm consisting of four hen houses with 30,000 birds in each house. Chicks were from Ross 308 parent flocks 40 weeks old. At the hatchery, during egg transfer (18 days and 9 h), different in ovo vaccines against IBDV were administered, and one group (36,000) of eggs was not vaccinated (control group). The experiment complied with Directive No. 2010/63/EU and the Act of 15 January 2015, protecting animals used for scientific or educational purposes. The research material and blood samples were obtained after the slaughter and routine veterinary work. The research follows the ARRIVE guidelines.

H1—Hen house I: Embryos were vaccinated in ovo during egg transfer at 18 days and 9 h with the IBDV vaccine containing the vHVT013-69 strain at a dose of 0.05 mL/embryo. Then, after hatching, chicks were vaccinated against infectious bronchitis virus (IBV) in the hatchery by spraying with a vaccine containing the H-120 strain (serotype Mass) and a vaccine containing the CR88121 strain (serotype 793B) at a dose of 20 mL/100 birds and also by spraying with a vaccine against NDV containing the VG/GA strain at a dose of 20 mL/100 birds.

H2—Hen house II: Embryos were vaccinated in ovo during egg transfer at day 18 and 9 h with IBDV vaccine containing Winterfield 2512 strain at a dose of 0.05 mL/embryo. Then, after hatching, chicks were vaccinated against infectious bronchitis virus (IBV) in the hatchery by spraying (droplet size, 120 µm) with vaccine containing H-120 strain (serotype Mass) and vaccine containing CR88121 strain (serotype 793B) at a dose of 20 mL/100 birds and also by spraying with NDV vaccine containing VG/GA strain at a dose of 20 mL/100 birds.

H3—Hen house 3: Embryos were vaccinated in ovo during egg transfer on day 18 and 9 h with IBDV vaccine containing M.B. strain at a dose of 0.05 mL/embryo. Then, after hatching, the chicks in the hatchery were vaccinated by spraying (droplet size, 120 µm) together against infectious bronchitis virus (IBV) with a vaccine containing the H-120 strain (Mass serotype) and a vaccine containing the CR88121 strain (serotype 793B) and vaccine against NDV containing the VG/GA strain at a dose of 20 mL/100 birds.

HK—Hen house 4: The chicks in this hen house were not vaccinated; this was the control group.

The birds were kept in windowless henhouses with artificial lighting (20 lux) and regulated environmental conditions on straw litter with unlimited access to water. At the beginning of rearing, the temperature in the henhouse was 32 °C, which was gradually reduced to 20–21 °C on day 21. Air humidity and ventilation were adjusted during daily observations, and the amount of harmful gases did not exceed the permissible standards: CO_2_—3000 ppm, NH3—20 ppm, and H2S—5 ppm. During rearing, which lasted up to 42 days, 3 feeding periods were used: from day 1 to 10 starter (22.7% crude protein and 12.3 MJ metabolizable energy (ME) in 1 kg of mixed feed), from day 11 to 35 grower (20.5% crude protein and 12.9 MJ ME), and from day 36 to 42 finisher (18.4% crude protein and 13.3 MJ ME).

At day 1, 21, 31, and 41, blood was collected for serological tests to determine the antibody titer against IBDV and NDV (n = 23). Samples were taken randomly from birds in three locations: at the beginning, in the middle, and at the end of the hen house. The samples were delivered to an accredited laboratory where a commercial ELISA kit (IDEXX NDV, IDEXX IBD, France; ID-Vet IBD VP2, France) (Thermo Fisher, Waltham, MA, USA) was used. The ELISA kits were used according to the manufacturer’s protocol. The antibody titer of each bird was quantified using a BioTek ELx800 reader (BioTek Instruments, Inc., Winooski, VT, USA) at a wavelength of 650 nm and the software provided by the manufacturer (BioCheck, software, version 2023.2). All serum samples were read against the provided antisera and positive and negative controls.

During the necropsy of the birds in an accredited laboratory on days 21, 31, and 41, the bursae of Fabricius were collected from 5 chickens and weighed individually on an electronic scale PS 1000.R2 (Radwag, Radom, Poland) with an accuracy of 0.01 g. Then, the bursae were fixed in 10% neutralized formalin. The bursae of Fabricius were fixed with 10% buffered formalin solution and then dehydrated with alcohol and xylene. The prepared samples were embedded in paraffin blocks. The blocks were cut into 5 µm thick pieces using a microtome and placed on glass slides. The slides were counterstained with hematoxylin and eosin. Then, the bursae were assessed on two occasions (21 and 31 days) using the following scale: 0—bursae without damage, 1—bursae with mild damage: samples of the bursa of Fabricius showing mild damage in the core of several follicles, 2—extensive: samples showing damage, hemorrhage, and necrosis of lymphoid cells of the follicles. Inflammation and cyst formation occur. The core and cortex of several follicles are indistinguishable, and the tissue between the follicles is edematous.

Tissue sections of the bursa of Fabricius were immunohistochemically stained with the BU1 antibody, labeled with 3,3′-diaminobenzidine (DAB), against chicken B cells. The slides were stained with hematoxylin. Digital photographs of each section were then taken, and a study area (swarm) of the same size (approximately 0.1 mm^2^) was defined. The study areas (swarms) were analyzed separately using HistoQuest 4.0.4.150 software for automatic quantification of cells based on their staining. Each detected cell was marked by the software: green as BU1 negative cells and red as BU1 positive cells (B cells).

The collected numerical data were described using generally accepted methods of mathematical statistics. Using the SAS package (version 9.4, SAS Institute, Cary, NC, USA), the mean values (x) and standard deviations (SD) of the studied traits were calculated; then, the effect of the experimental factor on the studied traits was examined using analysis of variance, and the significance of differences was evaluated using the Tukey test. The significance of differences between groups was verified at *p* < 0.05.

## 3. Results and Discussion

Due to the high morbidity and mortality of birds associated with infectious bursal disease, which occurs worldwide and causes economic losses, various preventive programs are used to protect birds against IBD virus [5,12]. According to the authors Thomrongsuwannakij et al. [12], the effect of vaccines against IBDV is immunosuppressive, causing an insufficient immune response (antibody level) against other diseases, such as NDV. The presented study compared three vaccines administered in ovo against IBDV on the immune response and their effect on vaccination against NDV in broiler chickens. The assessment of the bursa of Fabricius was also performed in terms of damage and the number of B lymphocytes. A significant (*p* < 0.05) effect of the vaccination programs used (Table 1) against IBDV on the level of antibodies in chickens expressed as geometric mean titer (GMT) was demonstrated.

Serum analysis using the IDEXX test showed significant differences between groups at three dates (21 d, 31 d, and 41 d). The highest GMT level on day 21 was demonstrated in the group of chickens vaccinated with the vaccine containing the vHVT013-69 strain and the control group (H4) (*p* = 0.008). On day 31, the group of chickens in which the vaccine containing the M.B. strain was used was characterized by the highest GMT, while the lowest GMT level was observed in the H1 (vHVT013-69 strain) and H4 (control) groups (*p* < 0.001). These results confirm the findings of other authors in previous studies [12,13]. Thomrongsuwannakij et al. [12] showed a higher level of GMT on days 36 and 43 in chickens in which the very virulent strain (M.B.) was used compared to the immune response (GMT) induced in chickens in which the vaccine containing the classical V217 strain was used. The authors showed that the use of vaccines containing more virulent strains induces a higher immune response expressed in GMT. In the presented studies, it was observed that at the end of the chicken rearing period (day 41), all three groups were characterized by a similar level of GMT, while a significantly lower level was shown in the control group (*p* < 0.001). The authors Sedeik et al. [13] showed the effect of the applied vaccination programs against IBD on GMT in chickens on days 21, 28, and 35. Śmiałek et al. [5] showed the effect of the applied vaccination programs on GMT at the end of the broiler chicken rearing period. The analysis of the geometric mean titer in the serum of broiler chickens using the vp2—ID-Vet test (Table 1) showed significant (*p* < 0.05) differences in the studied terms (days 31 and 41). The highest GMT was found in the serum of chickens in H2 and H3, while the lowest was in the control group (*p* < 0.001). At the end of rearing (day 41), the highest level of GMT in serum was characteristic of birds vaccinated with the vaccine containing the vHVT013-69 strain, while the lowest level was shown in chickens from the control group (*p* < 0.001). The vaccination program against IBDV applied, depending on the group of chickens, did not affect the level of GMT in the serum of the examined chickens against NDV in the analyzed dates. The mean geometric titer in the three vaccinated groups was at a similar level; only chickens from the control group were characterized by a significantly lower level of GMT (*p* = 0.026). Other authors showed different results [12]. In the group of chickens in which the vaccine containing the M.B. strain was used, the mean geometric titer on day 36 tested against NDV was significantly lower compared to the group of birds vaccinated with the vaccine containing the classical V217 strain. The authors concluded that the more virulence vaccine used resulted in lower titers expressed in GMT against NDV.

The use of attenuated vaccines, especially those containing very virulent strains, can cause damage to the bursa of Fabricius structures, which in turn leads to reduced B lymphocyte proliferation [5]. According to the authors, bursa damage most often occurs 2 to 4 weeks after vaccination; however, vaccines containing very virulent strains induce a faster immune response to protect chickens against IBD [5,12]. In the presented studies (Table 2), no significant differences were shown between groups in the weight of the bursa of Fabricius on day 21 (*p* = 0.148).

However, the vaccination program used affected the analyzed trait on days 31 and 41. The bursa weight was significantly higher in the groups (H1 and H4) compared to the BF weight from the H2 and H3 groups (*p* < 0.001). The lower bursa weight in the H2 and H3 groups was most likely due to the vaccines used, which contained live viruses that have a destructive effect on the BF. Since the vaccine used in H1 did not contain a live virus, only VP2 protein, and H4 was an unvaccinated control group, it did not affect the BF damage. These results confirm the findings of Rashid et al. [14], who also showed higher BF weights in the group of chickens vaccinated with the vaccine containing the vHVT013-69 strain compared to the other groups on days 26, 31, and 40 of rearing. Histological analysis, which is not presented in the table, showed damage to the bursa of Fabricius at the level of 0.2 points for H1; H2 and H3—2.0 points; and H4—0.0 points. In groups H2 and H3, the bursae showed losses, bleeding, and necrosis of the lymphoid cells of the follicles. Inflammation and cyst formation also occurred, and the tissue between the follicles was swollen. Other studies have also shown lower levels of BF forms in bird organizations with an additional module of recombinant form [14,15]. Rashid et al. [14] also showed differences between the level of BF damage depending on the vaccination program used on days 21, 28, and 35 in broiler chickens. The authors observed a lower level of damage in the group of birds vaccinated with a vaccine containing only the VP2 gene. Kajal et al. [16] showed that the BF damage was influenced by the vaccination date. Birds vaccinated with a single dose on day 17 had less BF damage compared to the group of birds that received a booster vaccination on day 24. On the other hand, Śmiałek et al. [5], using a vaccination program based on vaccines containing live IBD virus, showed differences in the level of BF damage in chickens at the end of the rearing period. The level of BF damage has a significant impact on the production of B lymphocytes. In the presented studies, it was shown (Table 2) that the vaccination program applied influenced the number of B lymphocytes, which was significantly (*p* < 0.05) higher in groups (H1 and H4) in which the vaccine containing the attenuated virus was not used (*p* < 0.001). Also, the total number of cells in H1 and H4 was significantly higher compared to H3 and H4 (*p* < 0.001). Cheng et al. [17] showed that the intestinal microbiota influences the development of B lymphocytes in the BF of young broilers. Antibiotic treatment led to a decrease in the number of Bu-1+ cells and a decrease in the levels of immunoglobulins IgA, IgM, and IgY in the BF at the end of rearing (day 49).

## 4. Conclusions

In summary, it can be stated that the use of vaccines containing attenuated virus in immunoprophylaxis of chickens, especially those that overcome the higher titer of maternal immunity, will result in a higher stimulation of the immune response and cause greater BF damage, which will consequently lead to lower production of B lymphocytes. The applied vaccination program against IBD did not influence the immune response of the immune system expressed in the GMT of the tested chickens against NDV.

## Figures and Tables

**Table 1:** Serum antibody titers IBD and ND in broilers at different ages.

Age	Group				SEM	*p*-Value
	**H1**	**H2**	**H3**	**H4**		
IBD—IDEXX						
1 d	5073.6 ± 1266.6	4771.9 ± 1353.5	4481.2 ± 1277.2	4147.0 ± 1165.4	134.90	0.088
21 d	300.5 ± 288.6 ^a^	120.7 ± 119.5 ^b^	138.7 ± 193.8 ^b^	615.4 ± 153.3 ^a^	21.50	0.008
31 d	447.3 ± 420.3 ^c^	3023.5 ± 717.1 ^b^	10,453.2 ± 2065.5 ^a^	227.5 ± 125.3 ^c^	441.39	<0.001
41 d	3029.3 ± 1077.9 ^a^	2569.0 ± 788.9 ^a^	2602.2 ± 815.4 ^a^	30.7 ± 37.7 ^b^	147.41	<0.001
ND—IDEXX						
1 d	8338.9 ± 1941.7	8745.5 ± 2021.7	8826.4 ± 1759.2	8229.2 ± 1718.1	199.81	0.099
21 d	470.6 ± 297.9	517.2 ± 671.2	568.7 ± 601.1	433.8 ± 282.9	51.04	0.811
31 d	199.7 ± 219.6 ^a^	176.7 ± 257.5 ^a^	180.6 ± 164.9 ^a^	45.4 ± 39.6 ^b^	20.45	0.026
41 d	161.4 ± 225.7	320.9 ± 863.9	165.4 ± 268.3	13.09 ± 15.5	49.15	0.179
IBDvp2—ID-Vet						
31 d	6964.0 ± 4668.5 ^b^	10,169.0 ± 1427.3 ^a^	10,453.2 ± 2065.5 ^a^	611.5 ± 153.4 ^c^	496.48	<0.001
41 d	10,379.8 ± 1751.7 ^a^	8075.8 ± 2070.9 ^b^	7918.0 ± 1897.4 ^b^	50.5 ± 35.6 ^c^	443.18	<0.001

Note: Values are expressed as means ± standard deviation, n = 23/group; ^a,b,c^ *p* < 0.05—means with different superscripts in the same row are statistically different between groups. Abbreviation: test ID-Vet Innovative Diagnostics protein detector vp2, IBDvp2—ID-Vet.

**Table 2:** Bursa of Fabricius weight and the number of B lymphocytes in chickens of different ages.

Item	Age	Group				SEM	*p*-Value
		**H1**	**H2**	**H3**	**H4**		
Bursa of Fabricius weight (g)	21 d	1.54 ± 0.46	1.14 ± 0.23	1.05 ± 0.38	1.34 ± 0.21	0.56	0.148
	31 d	2.69 ^a^ ± 0.70	0.75 ^b^ ± 0.12	0.67 ^b^ ± 0.17	2.79 ^a^ ± 1.27	0.16	<0.001
	41 d	1.77 ^b^ ± 0.75	0.77 ^c^ ± 0.14	0.61 ^c^ ± 0.17	2.63 ^a^ ± 0.76	0.44	<0.001
B lymphocyte count	41 d	17,152.8 ^a^ ± 9141.2	6423.4 ^b^ ± 887.9	5448.2 ^b^ ± 2910.8	20,882.8 ^a^ ± 1746.3	3675.33	<0.001
Total number of cells	41 d	23,136 ^a^ ± 2773.0	20,414.4 ^b^ ± 1121.4	19,792.2 ^b^ ± 456.0	24,213.2 ^a^ ± 1079.2	1073.67	<0.001

Note: Values are expressed as means ± standard deviation, n = 5/group; ^a,b,c^ *p* < 0.05—means with different superscripts in the same row are statistically different between groups.

## Data Availability

The original contributions presented in this study are included in the article. Further inquiries can be directed to the corresponding author.
